# Laparoscopic right hemicolectomy: a SICE (Società Italiana di Chirurgia Endoscopica e Nuove tecnologie) network prospective study on the approach to right colon lymphadenectomy in Italy: is there a standard?—CoDIG 2 (ColonDx Italian Group)

**DOI:** 10.1007/s00464-023-10607-8

**Published:** 2024-01-08

**Authors:** G. Anania, M. Chiozza, A. Campagnaro, F. Bagolini, G. Resta, D. Azzolina, G. Silecchia, R. Cirocchi, A. Agrusa, D. Cuccurullo, M. Guerrieri, SICE CoDIG (ColonDx Italian Group), V. Adamo, M. Ammendola, P. Angelini, M. Annecchiarico, G. Aprea, F. Autori, G. Baldazzi, A. Balla, G. Baronio, G. Bellio, G. Bertelli, C. Bima, L. D. Bonomo, D. Borreca, E. Botteri, A. Brescia, L. Cafagna, P. Capelli, V. Caracino, M. Caricato, M. Carlini, E. Cassinotti, M. Catarci, P. Chiaro, N. Cillara, M. Clementi, R. B. Contul, G. Curro, N. De Manzini, M. Degiuli, D. Delogu, A. Di Leo, U. Elmore, G. Ercolani, F. Festa, R. Galleano, G. Gambino, R. Gelmini, A. Giordano, R. La Mendola, L. Laface, L. Masoni, A. Maurizi, R. Memeo, P. Mercantini, G. Merola, M. Milone, M. Montuori, L. Morelli, I. A. Muttillo, R. Nascimbeni, T. Nelli, S. Olmi, M. Ortenzi, A. Patriti, G. Pavone, M. Pisano, R. Polastri, D. Rega, M. Rottoli, E Saladino, M. Santarelli, R. Santoro, A. Sartori, M. Scatizzi, G. Sica, W. Siquini, M. Sorrentino, F. Staderini, L. Vincentini, G. Aizza, M. Ammendola, P. Amodio, F. Aquilino, G. Argenio, A. Avanzolini, L. Baldari, F. Banchini, M. Benedetti, V. Bertino, A. Bianco, F. Blasi, L. Bonariol, D. Bono, A. Bottari, S. Buscemi, G. Calini, R. Campagnacci, S. Cantafio, G. T. Capolupo, M. Capuano, F. Carannante, M. Casati, D. Cassini, S. Castiglioni, C. Cecconi, L. Cestino, N. Chetta, F. M. Chiappetta, L. Cinelli, A. Cojutti, D. Colettta, D. Corallino, L. Crepaz, S. Curcio, G. Cuticone, F. D’Agostino, M. De Luca, G. D. De Palma, C. De Rosa, A. De Serra, R. Del Giudice, G. Di Franco, F. Foglio, G. Fontani, L. Fortuna, M. R. Fortunato, D. Frazzini, N. Furbetta, E. Gambino, I. Garosio, P. Germani, O. Ghazouani, D. Giannotti, E. Gibin, A. Grasso, M. Grieco, D. Izzo, G. G. Laracca, G. Lauteri, P. Lepiane, F. S. Li Causi, E. Locci, G. Lorenzo, A. Madaro, F. Madeddu, F. Maggi, F. Maiello, M. Manigrasso, R. Marcellinaro, P. Marinello, M. S. Mattei, G. Mazzarella, G. Merola, F. Moroni, A. Murgese, E. M. Muttillo, A. Oldani, M. Paicilli, M. Palmieri, G. Palomba, G. Paolini, D. Parini, G. M. Paroli, M. Pellicciaro, N. Petrucciani, B. Picardi, R. Piccolo, E. Pinotti, A. Pisanu, R. Reddavid, A. Resendiz, G. Romano, E. G. Rossi, R. Saracco, R. Scaramuzzo, F. Serra, E. Sgotto, L. Solaini, M. Spalluto, L. Taglietti, E. Tartaglia, N. Tartaglia, B. Torre, R. Tutino, M. Varesano, N. Vettoretto, E. Villamaina, T. Viora, M. Yusef, M. Zago, A. Zerbinati

**Affiliations:** 1 Unit of General Surgery, Department of Medical Science, University of Ferrara, via Fossato di Mortara 64/B, Ferrara, FE Italy; 2 Division of Scienze dell’Ambiente e della Prevenzione, University of Ferrara, Ferrara, FE Italy; 3 Department of Scienze Medico Chirurgiche e Medicina Traslazionale, University of Roma S. Andrea University Hospital, Rome, RM Italy; 4 Department of Medicine and Surgery, University of Perugia, Perugia, PG Italy; 5 Department of Surgical, Oncological and Oral Sciences, University of Palermo, Palermo, PA Italy; 6 Division of Laparoscopic and Robotic Surgery Unit, A.O.R.N. dei Colli Monaldi Hospital, Naples, NA Italy; 7 Division of Clinica Chirurgica Generale e D’Urgenza, Università Politecnica delle Marche, Ancona, AN Italy

**Keywords:** Right hemicolectomy, Ileocolic anastomosis, Laparoscopy, Lymphadenectomy, CME, Outcomes

## Abstract

**Background:**

Colon cancer is a disease with a worldwide spread. Surgery is the best option for the treatment of advanced colon cancer, but some aspects are still debated, such as the extent of lymphadenectomy. In Japanese guidelines, the gold standard was D3 dissection to remove the central lymph nodes (203, 213, and 223), but in 2009, Hoenberger et al. introduced the concept of complete mesocolic excision, in which surgical dissection follows the embryological planes to remove the mesentery entirely to prevent leakage of cancer cells and collect more lymph nodes. Our study describes how lymphadenectomy is currently performed in major Italian centers with an unclear indication on the type of lymphadenectomy that should be performed during right hemicolectomy (RH).

**Methods:**

CoDIG 2 is an observational multicenter national study that involves 76 Italian general surgery wards highly specialized in colorectal surgery. Each center was asked not to modify their usual surgical and clinical practices. The aim of the study was to assess the preference of Italian surgeons on the type of lymphadenectomy to perform during RH and the rise of any new trends or modifications in habits compared to the findings of the CoDIG 1 study conducted 4 years ago.

**Results:**

A total of 788 patients were enrolled. The most commonly used surgical technique was laparoscopic (82.1%) with intracorporeal (73.4%), side-to-side (98.7%), or isoperistaltic (96.0%) anastomosis. The lymph nodes at the origin of the vessels were harvested in an inferior number of cases (203, 213, and 223: 42.4%, 31.1%, and 20.3%, respectively). A comparison between CoDIG 1 and CoDIG 2 showed a stable trend in surgical techniques and complications, with an increase in the robotic approach (7.7% vs. 12.3%).

**Conclusions:**

This analysis shows how lymphadenectomy is performed in Italy to achieve oncological outcomes in RH, although the technique to achieve a higher lymph node count has not yet been standardized.

Trial registration (ClinicalTrials.gov) ID: NCT05943951.

Colon cancer is the second most common cancer in women and the third most common cancer in men [1].

The best option for treating advanced colon cancer without distant metastasis is surgery. However, the best surgical course of action is still under debate, with the extension of lymphadenectomy being one of the most contentious issues [2, 3].

While it has been demonstrated that the laparoscopic approach for colon cancer surgery is comparable to open surgery in terms of safety and oncological outcomes [4], the type of lymphadenectomy that must be performed does not have the same scientific certainty. Since the 1980s, for colon cancer with a stage higher than T2, Japanese guidelines have recommended removing the central lymph nodes (D3 dissection) during colectomy [5], but in 2009, Hohenberger et al. [6] introduced the concept of complete mesocolic excision (CME). They emphasized that surgical dissection must strictly follow the embryological planes to remove the diseased colon and its mesentery as a unit and prevent the leakage of cancer cells. To date, there have been several studies, with others in progress [7], comparing these two techniques. Recently, we have seen that the CME procedure harvests a larger area of the mesentery and more lymph nodes, but the duration of surgery is longer with CME than with D2 [2, 8, 9]. CME does not increase the intraoperative and postoperative complications of laparoscopic right hemicolectomy (RH); however, the risk of vascular injury during CME is significantly increased [2]. With the data now available, it is not possible to verify with certainty that CME has improved oncological survival; it has not been shown to be less feasible and less safe than standard surgery, but it is a more complex and difficult procedure, requiring more operative experience and a longer learning curve [10, 11].

Adding to these promising oncological benefits, the CME procedure offers increased accuracy for patient stadiation through removal and analysis of lymph nodes from stations that are not considered in traditional RH, provided increased possibilities of detecting nodal metastases that otherwise go unnoticed, as nodal spread does not always start from the stations more proximal to the primitive lesion [12, 13]. Our study aims to provide a picture of how lymphadenectomy is currently performed in many Italian centers in an international scenario where indications are mostly unclear, especially regarding the type of lymphadenectomy that should be performed during RH (Fig. 1).

**Fig. 1:**
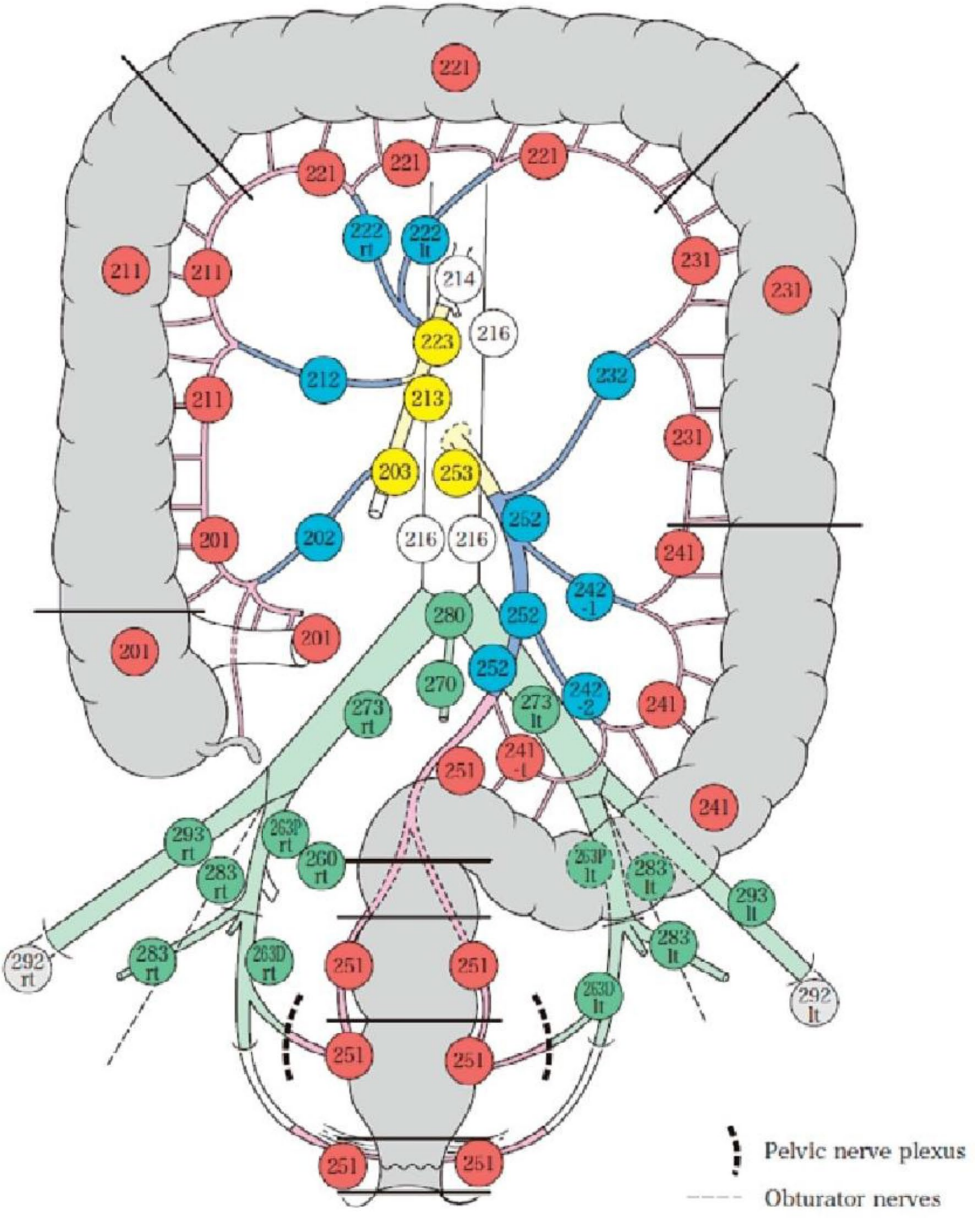
Lymph node locations. *Japanese Classification of Colorectal, Appendiceal, and Anal Carcinoma—3rd English edition, 2019*

## Materials and methods

### Study design

The CoDIG 2 (ColonDx Italian Group—Italian Right Colon Group) study was designed as a cohort, prospective, observational, multicenter national study comparing the risk of postoperative complications during RH to the extent of the lymphadenectomy that is performed. Patients were recruited over a 6-month period from April 2022 to October 2022. The study was approved by SICE (Società Italiana di Chirurgia Endoscopica e Nuove Tecnologie—Italian Society of Endoscopic Surgery and New Technologies). The Coordinator Center and Promoter of the study is the 1st Department of General Surgery of Ferrara University Hospital. The study involved 76 Italian general surgery centers that are highly specialized in colorectal surgery. Data were collected using the official SICE website database. Each center was asked not to modify their traditional surgical and clinical practices, including the technologies used, the surgical approach, the lymphadenectomies performed, and the pre- and postoperative management (including the ERAS, Enhanced Recovery After Surgery, protocol). Patients involved in the study signed an informed consent form. The primary endpoint of the study was to assess the preference of Italian surgeons on the type of lymphadenectomy to perform during RH and its results in terms of lymph node harvest. The secondary endpoint was to compare CoDIG 1 and CoDIG 2 in terms of surgical techniques used, anastomosis and postoperative outcomes to describe the evolution of right colon surgery in Italy after 4 years.

### Population and inclusion and exclusion criteria

Each center was committed to enrolling all consecutive patients observed during the study period aged > 18 years old who underwent elective laparoscopic or robotic RH.

The exclusion criteria were emergency surgery, laparotomic RH, ASA > IV, and pregnancy.

The online collection (SICE website database) of the data for each patient enrolled included the following:

- Patient characteristics: sex, age, BMI, Charlson comorbidity index.
- Indication for surgery: ASA score, preoperative diagnosis, TNM stage, histologic diagnosis.
- Intraoperative data: surgical technique, operative time, type of anastomosis, and intraoperative complications.
- Pathological anatomy information: tumor-free margin, terminal ileum length, vascular ligature, mesocolon sail integrity and area, and total lymph nodes/positive lymph nodes.
- Postoperative management: ERAS protocol adherence, postoperative hospital stay, postoperative complications.

A total of 788 patients were recruited by the 76 participating centers over a period of 6 months from April 2022 to October 2022.

### Complete lymphadenectomy vs. incomplete lymphadenectomy

To conduct a comparative analysis in a nonrandomized real-life study setting, a series of variables regarding the surgical technique of lymphadenectomy was used to divide the patients into two groups, one in which the basic principles of an allegedly correct lymphadenectomy were adhered to and one in which at least one factor was not adhered to. The factors considered were ligation at the origin of the ileocolic artery, ligation at the origin of the right branch of the medium colic artery, integrity of the mesocolic sail [3, 6] and tumor-free margin > 5 cm [14]. Two groups were then created: Group C (complete lymphadenectomy) included the patients in whom all variables were achieved, and Group I (incomplete lymphadenectomy) included the patients in whom one or more of these quality landmarks were not achieved.

### Statistical analysis

The data are expressed as the median and interquartile range for quantitative variables and absolute and relative frequencies for qualitative variables. To analyze differences between groups, the Wilcoxon test was utilized for numerical features, while the chi-square test or, if suitable, Fisher’s exact test was used for categorical features.

A multivariable gamma model was calculated for the overall number of lymph nodes, taking into account the skewed nature of the response variable. The impacts of unit increases were documented along with their 95% confidence intervals, achieved by calculating the marginal effect through a partial derivative of the expected value. Furthermore, a multivariable zero-inflated beta model was applied to the proportion of positive lymph nodes. This model addressed how the covariates influenced both the proportion of positive lymph nodes (beta regression) and the likelihood of encountering no positive lymph nodes out of the total count during the procedure (zero inflation).

The computations were performed with R 3.4.2 with the gamlss[1] and rms[2] packages.

## Results

Seven hundred eighty-eight patients were enrolled; 370 (47.0%) were male, and 418 (53.0%) were female, with an average age of 74 years. Regarding BMI, 1.78% had BMI scores < 18, 47% had BMI scores between 18 and 24, 38.2% had BMI scores between 25 and 30, and 13.1% had BMI scores over 30. The overall population had a median Charlson comorbidity score of 5 points. Previous abdominal surgery had occurred in 364 patients (46.2%). The indications for elective right hemicolectomy were benign disease in 39 cases (4.95%) and malignant disease in 749 cases (95.1%). According to the American Society of Anesthesiologists (ASA) score, 3.43% were ASA I, 45.6% were ASA II, 44.7% were ASA III, and 6.35% were > ASA III (Table 1).

**Table 1:** Patient characteristics

	Tot (788)	Complications	
		Yes (619)	No (169)
	*n* (%)		
Sex			
Male	370 (47.0%)	300 (48.5%)	70 (41.4%)
Female	418 (53.0%)	319 (51.5%)	99 (58.6%)
Age	74.0 [67.0;81.0]	74.0 [66.0;80.0]	76.0 [71.0;82.0]
BMI			
< 18	14 (1.78%)	13 (2.10%)	1 (0.59%)
18–24	370 (47.0%)	288 (46.5%)	82 (48.5%)
25–30	301 (38.2%)	240 (38.8%)	61 (36.1%)
> 30	103 (13.1%)	78 (12.6%)	25 (14.8%)
ASA score			
I	27 (3.43%)	25 (4.04%)	2 (1.18%)
II	359 (45.6%)	292 (47.2%)	67 (39.6%)
III	352 (44.7%)	271 (43.8%)	81 (47.9%)
> III	50 (6.35%)	31 (5.01%)	19 (11.2%)
Charlson comorbidity score	5.00 [3.00;6.00]	5.00 [3.00;6.00]	6.00 [4.00;7.00]
Previous abdominal surgery			
Yes	364 (46.2%)	284 (45.9%)	80 (47.3%)
No	424 (53.8%)	335 (54.1%)	89 (52.7%)
Pathology			
Benign	39 (4.95%)	35 (5.65%)	4 (2.37%)
Malignant	749 (95.1%)	584 (94.3%)	165 (97.6%)

### Surgical techniques

The most commonly used surgical technique was laparoscopic in 82.1% of patients, and alternative robotic surgery and video-assisted techniques were performed in 11.5% and 6.35% of patients, respectively. Robotic surgery involves the use of a robotic device, while video-assisted surgery refers to a hybrid laparoscopic technique with anastomosis performed through service access. Regarding operating time, less than 180 min was needed in 52.3% of patients, 180–270 min in 37.2%, and more than 270 min in 10.5%. Blood transfusion was necessary in 28 patients (3.55%). ICA was performed in 578 (73.4%) patients, while ECA was performed in 210 (26.6%) patients. Side-to-side (98.7%) and isoperistaltic (96.0%) anastomosis were the most common techniques. Mesocolon closure was performed in less than half of patients (48.0%), with interrupted sutures in 87 patients and continuous sutures in 291 patients (11.0% and 36.9% of all patients, respectively). Among alternatives to specimen extraction, a Pfannenstiel incision was most common at 53.0%, followed by a median upper umbilical incision at 25.1%. The rate of conversion was 7.49% (Table 2).

**Table 2:** Descriptive analysis of intervention variables and surgical techniques

Variables	Type	*n* (%)
Surgical technique	Laparoscopic	647 (82.1%)
	Robotic	91 (11.5%)
	Video assisted	50 (6.35%)
Surgery time	> 270 min	83 (10.5%)
	181–270 min	293 (37.2%)
	90–180 min	412 (52.3%)
Need for blood transfusion	No	760 (96.4%)
	Yes	28 (3.55%)
Anastomosis	Intracorporeal (ICA)	578 (73.3%)
	Extracorporeal (ECA)	210 (26.7%)
Tailoring	L–L	778 (98.7%)
	Other	10 (1.27%)
Direction	Isoperistaltic	747 (96.0%)
	Anisoperistaltic	31 (3.98%)
ICA	Manual	26 (3%)
	Mechanical	836 (97%)
ECA	Manual	122 (33.6%)
	Mechanical	241 (66.4%)
Enterotomy closure	Manual	722 (91.6%)
	Mechanical	66 (8.38%)
Enterotomy manual closure	Single layer	111 (15.4%)
	Double layer	611 (84.6%)
Mesocolon closure	No	410 (52.0%)
	Yes, interrupted suture	87 (11.0%)
	Yes, continuous suture	291 (36.9%)
Specimen extraction incision	Pfannenstiel	418 (53.0%)
	Median subumbilical	56 (7.11%)
	Median upper umbilical	198 (25.1%)
	Transverse right hypochondrium	40 (5.08%)
	Other	76 (9.64%)
Conversion	Yes	59 (7.49%)
	No	729 (92.5%)

### CoDIG 1 vs. CoDIG 2

Some variables were compared between CoDIG 1 [15] and CoDIG 2. Regarding the surgical technique, there was a slight reduction in the laparoscopic technique in favor of the robotic approach (laparoscopic 92.3% vs. 87.7%; robotic 7.7% vs. 12.3%). Regarding anastomosis, an increase of 3% in intracorporeal anastomosis (ICA), which is mainly performed with mechanical staplers, was observed, confirming the previous data of CoDIG 1 [15]. The results of intraoperative and postoperative complications are similar, with a relative increase in intraoperative complications in CoDIG 2. The comparison of comparable data after 4 years between the two studies shows a stable trend in surgical technique and complications, with the exception of the robotic approach, which has been increasing in recent years (Table 3).

**Table 3:** Comparison of CoDIG 1 vs. CoDIG 2

Variables	Type	CoDIG 1	CoDIG 2
Technique used	Laparoscopic	1131 (92.3%)	647 (87.7%)
	Robotic	94 (7.7%)	91 (12.3%)
Anastomosis	ICA	862 (70.4%)	578 (73.3%)
	ECA	363 (29.6%)	210 (26.7%)
ICA	Manual	26 (3%)	22 (3.8%)
	Mechanical	836 (97%)	556 (96.2%)
ECA	Manual	122 (33.6%)	81 (38.6%)
	Mechanical	241 (66.4%)	129 (61.4%)
Intraoperative complications		20 (1.6%)	24 (4.2%)
	Intraperitoneal hemorrhages	(1.3%)	(1.6%)
	Iatrogenic small bowel lesion	(0.3%)	(1.2%)
Postoperative complications	Anastomotic bleeding	(4%)	(3.5%)
	Anastomotic leakage	(2.2%)	(2.8%)
	Bowel obstruction	(1.7%)	(3.7%)
	Intra-abdominal abscess	(1.8%)	(3.8%)
	Wound infection	(4.3%)	(4.4%)

### Lymphadenectomy

A tumor-free margin greater than 5 cm was achieved in most patients (89.9%), while a terminal ileum > 10 cm was achieved in only 43.4% of patients. Ligation at the origins of the colic vessels was preferred in 96.9% of patients for the ileocolic artery, 70.1% of patients for the right colic artery, and 67.8% of patients for the right branch of the medium colic artery. The integrity of the mesocolic sail was preserved in 88.3% of patients. The average number of harvested lymph nodes was 23. According to the Benz score [16], most specimens were type I (49.4%), followed by type 0 (37.6%), type II (11.2%) and type III (1.8%) (Table 4).

**Table 4:** Lymphadenectomy

Tumor-free margin	< 5 cm	79 (10.1%)
	> 5 cm	709 (89.9%)
Terminal ileum	< 10 cm	446 (56.6%)
	> 10 cm	342 (43.4%)
Ligation at the origin of the ileocolic artery	Yes	764 (96.9%)
	No	24 (3.1%)
Ligation at the origin of the right colic artery	Yes	552 (70.1%)
	No	236 (29.9%)
Ligation at the origin of the right branch of the medium colic artery	Yes	534 (67.8%)
	No	254 (32.2%)
Integrity of the mesocolic sail	Yes	696 (88.3%)
	No	92 (11.7%)
Number of lymph nodes harvested	Average	23
Number of positive lymph nodes	Average	1.45
Benz score [16]	Type 0	297 (37.6%)
	Type I	389 (49.4%)
	Type II	88 (11.2%)
	Type III	14 (1.8%)

Pathologist reports were consulted to identify the lymph node areas that were most commonly collected during RH surgery. Stations 201, 211 and 221, which are the most proximal to the colonic wall lymph node stations, were more frequently collected (87.9%, 86.2%, and 68.9% of cases, respectively). Similarly, the lymph nodes along the colic vessels (202, 212, and 222; 88.2%, 81.5%, and 64.7%, respectively) were among the most frequently found in specimens, while the lymph nodes at the origins of the vessels were harvested in an inferior number of cases (203, 213, and 223; 42.4%, 31.1%, and 20.3%, respectively) (Table 5).

**Table 5:** Areas of lymphadenectomy

201	693 (87.9%)
202	695 (88.2%)
203	334 (42.4%)
211	679 (86.2%)
212	642 (81.5%)
213	245 (31.1%)
221	543 (68.9%)
222	510 (64.7%)
223	160 (20.3%)

### Complete lymphadenectomy vs. incomplete lymphadenectomy

According to the reported data, in Italy, ligation at the origins of the ileocolic and right branch of the middle colic arteries, tumor-free margins > 5 cm and integrity of the mesocolic sail (Group C) are achieved in 55% of cases compared to 45% of cases in which at least one of these variables is not met (Group I). The stratified sample was homogeneous in terms of variables such as sex, age and BMI, with no statistically significant differences. Surgical technique also showed no statistically significant differences in distribution among laparoscopic, robotic and video-assisted procedures in the two groups (Table 6).

**Table 6:** Group C vs. Group I

No	Total	Group C	Group I	*p* value
	*N* = 749	*N* = 412 (55%)	*N* = 337 (45%)	
Sex				0.619
F	347 (46.3%)	187 (45.4%)	160 (47.5%)	
M	402 (53.7%)	225 (54.6%)	177 (52.5%)	
Age	74.0 [67.0;81.0]	74.0 [67.0;81.0]	75.0 [68.0;82.0]	0.304
BMI				0.913
BMI < 30	14 (1.87%)	7 (1.70%)	7 (2.08%)	
BMI > 30	735 (98.1%)	405 (98.3%)	330 (97.9%)	
Surgical technique				0.174
Laparoscopic	610 (81.4%)	328 (79.6%)	282 (83.7%)	
Robotic	90 (12.0%)	51 (12.4%)	39 (11.6%)	
Video assisted	49 (6.54%)	33 (8.01%)	16 (4.75%)	
Blood loss (ml)	70.0 [30.0;120]	77.5 [40.0;130]	60.0 [30.0;100]	0.114
Number of lymph nodes removed	22.0 [16.0;27.0]	22.0 [17.0;28.0]	21.0 [15.0;26.0]	0.002
Lymph nodes removed positive	0.00 [0.00;1.00]	0.00 [0.00;2.00]	0.00 [0.00;1.00]	0.183
Number of positive lymph nodes	252 (33.6%)	152 (36.9%)	100 (29.7%)	0.045
Postoperative complications (< 30 days)	165 (22.0%)	79 (19.2%)	86 (25.5%)	0.046
pTNM stage [American Joint Committee on Cancer (AJCC) Ed. IX]				0.011
I	200 (26.7%)	92 (22.3%)	108 (32.0%)	
II	277 (37.0%)	161 (39.1%)	116 (34.4%)	
III–IV	272 (36.3%)	159 (38.6%)	113 (33.5%)	
Benz score [16]				< 0.0001
Type 0	283 (37.8%)	201 (48.8%)	82 (24.3%)	
Type I	372 (49.7%)	191 (46.4%)	181 (53.7%)	
Type II	80 (10.7%)	18 (4.3%)	62 (18.4%)	
Type III	14 (1.8%)	2 (0.5%)	12 (3.6%)	

Benign lesions were excluded

Regarding the estimated intraoperative blood loss, there was no statistically significant difference between Groups C and I [Group C: 77.5 ml (40.0;130); Group I: 60.0 ml (30.0;100) *p*: 0.114].

The difference in both the number of lymph nodes harvested [Group C: 22.0 (17.0;28.0); Group I: 21.0 (15.0;26.0); *p*: 0.002] and the positive lymph nodes found on anatomopathological examination [Group C: 152 (36.9%); Group I: 100 (29.7%) *p*: 0.045] between the two groups was statistically significant.

We observed an increase in the number of registered complications at 30 days in Group I compared to Group C [Group C: 79 (19.2%); Group I: 86 (25.5%); *p*: 0.046] Finally, stratification by stage showed that a more complete procedure (Group C) was preferred in the more advanced stages (Stage III–IV: Group C, 38.6% vs. Group I, 33.5%; Stage II: Group C, 39.1% vs. Group I, 34.4%; *p* value 0.011) compared to the less advanced stages, where the less complete procedure was preferred (Group I, 32% vs. Group C, 22.3%; *p* value 0.011).

The correct division of patients was also confirmed by comparing the completeness of the surgical procedure with the Benz score, which showed a greater number of patients with extensive resection of the mesocolon in Group C (Type 0: Group C, 48.8% vs. Group I, 24.3%; *p* value < 0.0001) and a greater number of patients with less extensive resection in Group I (Type II: Group C, 4.3% vs. Group I, 18.4%; Type III: Group C, 0.5% vs. Group I, 3.6%; *p* value < 0.0001).

## Discussion

In recent years, since the number of recovered lymph nodes has been determined to significantly affect oncological prognosis, the extent of lymph node dissection during RH has attracted the attention of many colorectal surgeons [17] and has been a controversial point of discussion. This is due to a lack of agreement over the appropriate use of D2, D3 or CME dissection, which are often used interchangeably in the evaluation of anatomical landmarks and oncological qualities [18–20]. Since a compromise between increased morbidity and survival must be carefully considered, a standardized approach to choose among these procedures is becoming increasingly necessary [17]. In addition, a common standardized evaluation of the surgical area in relation to the completeness of CME is also necessary; the classification proposed by Benz et al., which we used in our study, could be a useful tool [16]. To provide more evidence to settle this debate, the Scientific Committee of SICE performed this observational prospective cohort study to collect data and provide a picture of how lymphadenectomy is currently performed in major Italian colorectal centers. The results of the survey demonstrated that the large majority of the Italian surgeons in this study have tried to achieve an oncologically correct lymphadenectomy by leaving a tumor-free margin greater than 5 cm, performing ligation at the origins of the colic vessels and maintaining the integrity of the mesocolic sail [21]; the achievement of all four variables in the same patient (Group C) is obtained in 55% of all right hemicolectomies.

In the two groups, there were no obvious differences in surgical technique (laparoscopic, robotic, or video-assisted) or amount of blood loss. A greater number of lymph nodes were harvested in Group C [Group C: 22.0 (17.0;28.0); Group I: 21.0 (15.0;26.0); *p*: 0.002], and more positive lymph nodes were found on anatomopathological examination [Group C: 152 (36.9%); Group I: 100 (29.7%) *p*: 0.045]. These results confirm the efficacy in lymph node harvesting during more invasive and complete lymphadenectomy compared to less aggressive lymphadenectomies. In addition, procedures with a complete lymphadenectomy are more widely used in oncologic disease with the more advanced pTNM Stages II and III–IV than in Stage I, in which lymphadenectomy is less likely to meet all criteria. These data show the different extent of lymphadenectomy depending on the stage of disease in a real-life scenario and how the disease stage influences the surgeon’s choices, tending to opt for a more complete lymphadenectomy in more advanced stages. However, 30-day postoperative complications were higher in Group I [Group C: 79 (19.2%); Group I: 86 (25.5%); *p*: 0.046]. This unexpected finding may be explained by the assumption that more invasive procedures are performed by more experienced surgeons with complete learning curves and are less prone to complications. The correct division of patients was also confirmed by the analysis of the anatomopathological results according to the Benz score, which showed a higher quality in Group C than in Group I.

The centers involved in the study requested data on which lymph node stations were collected during surgery, and lymph node stations along the axis of the superior mesenteric vessels were found to be the least frequently collected (203, 213, and 223; 42.4%, 31.1%, and 20.3%, respectively). This finding highlights that the role of central lymphadenectomy, which is riskier from the point of view of surgical safety, is still controversial; in fact, many Italian surgeons do not consider this procedure necessary to ensure complete lymphadenectomy, despite adhering to the principles used in our evaluation and indicated by the Benz score. Randomized studies on the benefits of removing the most central lymph node stations in both stadiation and therapy and the impact on patient survival are needed to increase understanding and to standardize and unify the procedure.

The data concerning the surgical technique showed a more widespread use of robotic technology and ICA 4 years after the first CoDIG study, outlining this direction for the future.

## Conclusion

This multicentric prospective observational study, with its inherent limitations, did not aim to define the standard of lymphadenectomy during RH but attempted to obtain a picture of how lymphadenectomy is performed in Italy and the direction of RH as a surgical procedure.

In 55% of cases, a complete lymphadenectomy (a lymphadenectomy that includes ligation at the origin of the colic vessels, maintenance of the mesocolic sail and disease-free resection margins > 5 cm) is performed, and this results in greater numbers of lymph nodes excised and positive lymph nodes. This choice is guided by the stage of disease, with surgeons showing a preference for more complete lymphadenectomies in more advanced stages.

While surgeons lean toward an extended lymphadenectomy for right colon cancer, the technique to achieve a higher lymph node count has not yet been standardized. RCTs, many of which are now ongoing, are needed to identify a common direction on the type of lymphadenectomy to be performed.
